# Patient participation during primary health‐care encounters among adult patients with multimorbidity: A cross‐sectional study

**DOI:** 10.1111/hex.13306

**Published:** 2021-07-10

**Authors:** Leila Paukkonen, Anne Oikarinen, Outi Kähkönen, Helvi Kyngäs

**Affiliations:** ^1^ Research Unit of Nursing Science and Health Management University of Oulu Oulu Finland; ^2^ Medical Research Centre Oulu Finland; ^3^ Oulu University Hospital Oulu Finland

**Keywords:** adherence, multimorbidity, multiple chronic conditions, patient activation, patient participation, patient preference, perceived health, primary health care

## Abstract

**Background:**

Patient participation is essential for achieving high‐quality care and positive outcomes, especially among patients with multimorbidity, which is a major challenge for health care due to high prevalence, care complexity and impact on patients' lives.

**Objective:**

To explore the patient participation related to their own care among patients with multimorbidity in primary health‐care settings.

**Methods:**

A cross‐sectional survey was conducted among adult multimorbid patients who visited primary health‐care facilities. The key instrument used was the Participation in Rehabilitation Questionnaire. Data representing 125 patients were analysed using various statistical methods.

**Results:**

The respondents generally felt patient participation to be important, yet provided highly varying accounts regarding the extent to which it was realized by professionals. *Information and knowledge* and *Respect and encouragement* were considered the most important and best implemented subcategories of participation. Several patient‐related factors had a statistically significant effect on patient perceptions of participation for all subcategories and as explanatory factors for perceptions of total participation in univariate models. Most patients reported active participation in health‐care communication, positively associated with patient activation and adherence. Gender, perceived health, patient activation and active participation were explanatory factors for total importance of participation in multivariate models, while patient activation was retained for realization of participation.

**Conclusions:**

Multimorbid patients require individualized care that promotes participation and active communication; this approach may further improve patient activation and adherence. Poor perceived health and functional ability seemed to be related to worse perceptions of participation.

**Patient and public involvement:**

The study topic importance was based on the patients' experiences in author's previous research and the need to develop patient‐centred care.

## 1 INTRODUCTION

The patient's role has changed significantly over the last few decades to a point where a patient is assumed to be an active partner rather than a passive recipient of care. In many Western countries, a patient's rights are also defined by legislation, policies and ethical principles. Various factors, such as movement away from paternalism, an emphasis on individual rights and democratic intentions, along with rising health‐care costs and the increasing burden of chronic conditions, have been suggested to underlie this shift.^1^, ^2^

Patient participation (PP) emphasizes patient's possibilities to get involved in and affect own care in partnership with health‐care providers (HCPs).^3^ It is important to note that PP is a broad and multidimensional concept with no single general definition of it,^4^, ^5^, ^6^, ^7^, ^8^ and many close and parallel concepts are also used interchangeably^9^, ^10^, ^11^ to describe how patients can become protagonists in their own health care.^12^ However, the generally recognized attributes of PP are established collaborative relationship, exchanges of information, knowledge and power, and mutual engagement in diverse activities, such as treatment planning and decision making between the patient and HCPs.^3^, ^4^, ^5^, ^7^ PP, when it is person‐centred and ideal, is based on patients’ experiences, values, preferences and needs with respect and equality as central issues. In health‐care encounters, mutual communicational elements are integral to PP.^3^ A patient's way of communicating in health‐care consultation has been considered to reflect his or her own active participation. This active participation manifests as asking questions (information seeking), expressing opinions, preferences and views (assertive utterances, expression of concern), and the provision of information.^13^, ^14^, ^15^

PP inherently involves social interaction; thus, all of the participants (eg patients, HCPs and organizations) influence its process. Previous studies in various health‐care settings have found that PP may be influenced by patient‐related factors such as sociodemographic factors, health state, perceived ability and perceptions about the adopted or expected role.^11^, ^16^, ^17^ Patients’ preferences may well vary from one treatment situation to another; for example, it is possible that some patients will not desire an active role in decision making, yet still value information that is relevant to their treatment.^3^, ^16^, ^17^, ^18^ Patients have also reported that HCPs can behave in a way that limits their participation, that is paternalistic attitude or the lack of individual recognition.^3^, ^11^, ^19^ Therefore, PP is a complex, multidimensional phenomenon that is not simple to implement in everyday health care^3^, ^19^ However, PP is valuable as it has been found to benefit both patients and the results of care by enhancing patient satisfaction and empowerment,^5^, ^17^ improving a patient's ability to accept responsibility^20^ and engage in self‐management activities,^16^, ^20^ and preventing medical errors to increase patient safety.^16^

PP is especially important in patients with chronic conditions, which can be controlled but not cured. The coexistence of two or more chronic conditions, called multimorbidity,^21^, ^22^ further emphasizes PP as a key component of effective and quality care. Studies have shown that both HCPs^23^, ^24^, ^25^, ^26^ and patients^25^, ^26^, ^27^, ^28^ find managing multimorbidity to be challenging. Multimorbid patients are a heterogeneous group with large variations in their physical and mental condition, as well as variation in how the conditions impact them. Furthermore, multimorbidity causes clinically complex cases due to interactions between diseases and medications. As such, health‐care professionals are tasked with addressing and prioritizing multiple, and possibly competing, conditions within a short time frame.^24^, ^29^ The clinical guidelines that HCPs commonly rely on to make appropriate care decisions are largely created for single conditions, and rarely address multimorbidity.^30^, ^31^ Multimorbid patients often receive fragmented care because they usually require care from distinct specialists for each condition, and these specialists may work at various health‐care organizations.^24^, ^29^ Studies mapping the experiences of multimorbid patients have identified several difficulties associated with this condition, for example contradictory ^32^ or insufficient information about their conditions and treatment options,^27^, ^28^, ^32^ and the lack of holistic care, guidance^28^ and decision‐making support,^26^ along with poor communication,^26^, ^27^, ^28^ coordination^26^, ^33^, ^34^ and continuity of care.^28^, ^34^ In addition, the patient and HCP do not always share a common view about care and health outcome priorities.^35^, ^36^ The self‐management required for everyday tasks is burdensome for most multimorbid patients; as such, many^37^ will need extensive support for motivation^25^ and understanding their own health situation.^26^, ^28^, ^32^, ^37^ This complexity may partly explain why multimorbidity is linked with an increased risk of patient safety incidents.^21^, ^38^, ^39^ These complicated health‐care needs highlight the need of more patient participation. Indeed, the patient‐centred, individualized approach is widely recognized as the best way to meet the care challenges posed by multimorbidity,^21^, ^29^, ^40^, ^41^ along in combination with integrative care,^42^, ^43^, ^44^ also suitable to primary care.^21^, ^29^, ^41^

Multimorbidity is common worldwide. Approximately one‐fourth of the population, and a majority of those aged 65 years or older have multimorbidity, and the prevalence of this condition is expected to increase.^45^, ^46^ Patients with multimorbidity use health‐care services more frequently—in terms of total number and number of specialized services—than patients without multiple chronic conditions.^46^, ^47^, ^48^, ^49^, ^50^ Multimorbidity imposes an especially heavy workload on primary health care,^48^, ^50^, ^51^ where it is present in most consultations ^48^ and increases hospital visits and the length of hospitalization.^47^ Multimorbid patients are also more likely to require emergency and/or secondary care than other patients.^49^ Multimorbidity is associated with negative health consequences, for example reduced functional status^49^, ^52^, ^53^ and decreased quality of life,^52^, ^54^ along with increased treatment burden,^51^, ^55^ polypharmacy^38^, ^40^ and premature mortality.^56^ Therefore, multimorbidity management has also considerable financial implications to health‐care systems.^47^, ^50^, ^57^

In the light of the increasing incidence of multimorbidity in the general population, and the impact of this condition on the health‐care process and outcomes, it would be important to study PP among multimorbid patients, especially from the patient's perspective. To the best of our knowledge, PP among multimorbid patients has only been studied to a limited extent. The aim of the study was to explore PP related to their own care in multimorbid patients in primary health‐care settings.

The specific research questions were as follows:

1. What are patients’ perceptions of the importance and the extent to which the PP was realized by professionals?
2. To what extent did patients themselves participate in health‐care encounter communication (=active participation)?
3. How do patient‐related factors influence patients’ perceptions of PP and active participation?
4. Which factors explain patients' perceptions of the importance and realization of PP?

## 2 METHODS

### 2.1 Study design, setting and participants

Data were collected using a cross‐sectional survey, which was implemented across all primary health centres in one Finnish municipality with about 200 000 inhabitants. The participants were adult multimorbid patients who visited a health‐care centre for chronic condition management between November 2019 and May 2020. The inclusion criterion was the coexistence of two or more chronic conditions, which fall under the following classifications: a long‐term physical, non‐communicable disease (eg cardiovascular disease, diabetes or cancer); a long‐term mental health condition (eg depression); or a long‐term infectious disease such as HIV or hepatitis C.^22^ Participants were also required to be at least 18 years of age and have sufficient Finnish‐language skills to complete a questionnaire. Sample size was calculated based on the previous information of PPRQ,^58^, ^59^ considering alpha = 0.05, power = 0.80 and effect size = 0.50 revealed that the minimum sample needed is 102 patients.

### 2.2 Data collection procedure

Prior to data collection, the service managers of the participating health centres—who would later distribute information about the study within their own units—were briefed on the study. Recruitment of study participants was performed by HCPs and took place during appointments with a nurse or doctor for the management of a chronic condition. Personnel were instructed to distribute questionnaires to all patients satisfying the eligibility criteria. The questionnaires included detailed written information about the study purpose and objectives, as well as the researchers’ contact information and a return postal envelope. Patients could complete the questionnaires at home and were asked to return the questionnaire within two weeks.

### 2.3 Measurements

**Patients' perceptions of the PP** were measured using the Participation in Rehabilitation Questionnaire (PPRQ). The original PPRQ was developed for patients with spinal cord injury to measure their perceptions of the importance and the degree to which PP was realized by professionals.^60^ Since then, the instrument has been validated and used in varied contexts.^58^, ^61^ The instrument includes five subscales: *Respect and integrity; Planning and decision making; Information and knowledge; Motivation and encouragement;* and *Involvement of family*. Respondents rate each item in terms of perceived importance and how frequently it was realized during their own care. Respondents are instructed to assess their care as a whole and refer to all personnel involved in their care (ie doctors, nurses, physiotherapists and psychologists). The provided response options are on a 5‐point Likert scale, namely, ‘not at all important’, ‘slightly important’, ‘important’, ‘very important’ and ‘extremely important’ (for importance), and ‘never’, ‘seldom’, ‘sometimes’, ‘often’ and ‘always’ (for realization). Respondents evaluate the importance and realization of PP separately.^60^ The modified Finnish version of the PPRQ used in this study consists of 19 items covering the same five scales as the original questionnaire. Details of the translation of the instrument and its validation in Finland are presented elsewhere.^58^ The mean score for each subscale was calculated as the average of valid values. However, to maintain validity, no value was entered if the respondent answered fewer than half of the items on the subscale. Cronbach's alpha coefficients in this study were 0.91 for importance ratings and 0.95 for realization ratings. Cronbach's alpha coefficients for the subscales ranged from 0.70 to 0.85 for importance and from 0.90 to 0.93 for realization.

The approach for evaluating **active participation** was derived from previously published studies and based on the extent to which patients ask questions and express their views/opinions^13^, ^14^, ^15^ Respondents were asked to assess their behaviour in health‐care encounters regarding their condition and care with two single statements: I ask questions (about the things I want to know, I do not understand, that need clarification, etc); and I express my views/opinions. Respondents answered these questions using a 4‐point Likert scale ranging from totally disagree to totally agree.

**Patient activation** was measured using the Patient Activation Measure (PAM‐13®),^62^ which is widely used for different chronic conditions and also validated in the context of multimorbid older adults.^63^ PAM includes 13 statements concerning the patient's knowledge, skills and confidence in managing their own health, as well as the belief in the importance of their own role. Respondents judge each item using a 4‐point Likert scale ranging from ‘strongly disagree’ to ‘strongly agree’, with an additional ‘not applicable’ option. The total score for all of the items (range: 13‐52) is then converted into a PAM score (range: 0‐100, with higher scores indicating higher activation) that can be categorized into one of four progressively higher levels of activation. Levels 1, 2, 3 and 4 correspond to scores of <47.1, 47.1 to 55.1, 55.2 to 67.0 and >67.1, respectively. These levels can also be used as cut‐offs.^62^, ^64^ The present study used the Finnish‐language version of PAM, available under licence from Insignia Health (Portland, OR, USA). According to their guidelines, respondents must answer 10 to 13 questions (N/A responses are considered missing) to obtain a valid PAM score. Because PAM is a Guttmann‐like scale characterized by increasing difficulty as the survey progresses, uniform response patterns should be considered unreliable and were therefore excluded from this study. In this study, the activation levels were dichotomized into low (levels 1 and 2) and high (levels 3 and 4) activation levels in accordance with previous studies.^65^ Cronbach's alpha value calculated in this study (0.84) indicates that the PAM instrument exhibits good internal consistency.

**Adherence to care** was assessed using the Finnish version of Adherence of people with Chronic Disease Instrument (ACDI),^66^ which has previously been applied to various chronic diseases^67^ and frequent health‐care attenders.^68^ This instrument includes 11 items that cover adherence to medications, care regimens, diet, monitoring, co‐operation, responsibility and willingness. Respondents answer each item using a 4‐point Likert scale ranging from ‘strongly disagree’ to ‘strongly agree’. For some of the performed analyses, the mean sum variables were categorized into three classes: poor (<3), adequate (3‐3.49) and good (≥3.5). Cronbach's alpha calculated for this instrument in the present study was 0.75.

**Chronic conditions constituting multimorbity:** The questionnaire contained a list of 26 distinct chronic conditions and an open‐ended question through which respondents could list additional chronic diseases not included in the provided list. The suitability of additional conditions was checked before they were included in the patient's total number of conditions. The respondents were asked to provide their height and weight for BMI and, subsequently, obesity calculations.

**Perceived health** was measured by the universally used indicator ‘How is your current health in general?’^49^, ^69^ The answer options were as follows: ‘good’, ‘quite good’, ‘moderate’, ‘quite poor’ and ‘poor’. These options were consistent with a study on health and functional capacity performed by the Finnish Institute for Health and Welfare.^70^

**Perceived functional ability** describes a patient's subjective experience of his or her ability to cope with meaningful and necessary daily‐life activities. This was assessed through one question: How is your current functional ability in general? The answer options were as follows: ‘good’, ‘quite good’, ‘moderate’, ‘quite poor’ and ‘poor’.^71^

The following **sociodemographic variables** were recorded for all respondents: year of birth; gender; marital status; highest educational level obtained; employment status; and living situation (alone, with spouse/children, etc).

### 2.4 Data analysis

Descriptive statistics were used to describe all of the variables and the sample characteristics. Means, standard deviations (SD) and ranges were used to describe continuous variables, whereas frequencies, percentages and their distributions were used for categorical variables. Visual inspection of the data (ie histograms and boxplots) and tests of normality were used to evaluate outliers and whether the data showed a normal distribution. Data obtained from the questionnaires were also classified for some analyses.

The main outcomes were patients’ perceptions of PP (measured by PPRQ) and patients’ active participation (manner of asking questions and expressing views/opinions). The latter were also used as independent variables for PPRQ scores. Independent‐samples t tests (pairwise comparisons) and one‐way analysis of variance (ANOVA; three or more groups) were used to assess the statistical significance of differences in mean PP between groups constructed for each independent variable studied. Differences between groups were calculated for each subscale concerning the importance and realization of patient participation separately. When differences in active participation were assessed, a chi‐square test (χ^2^ test) or Fisher‐Freeman‐Halton exact test was used for categorical variables, while a Kruskal‐Wallis test or ANOVA was used for continuous variables. A general linear model was used to examine which factors explain patients’ perceptions of patient participation. First, a univariate model was calculated for each explanatory variable and the total score of both importance and realization of patient participation. This was done to determine univariate associations and was not used as a selection method for candidate variables for multivariate models. Explanatory variables were age, gender, education, number of conditions, perceived health, perceived functional ability, patient activation, adherence and active participation. Then, all variables were integrated into a multivariate model. Because certain between‐variable correlations were observed, two multivariate models were presented for both dimensions of patient participation.

For all analyses, *P* < 0.05 was considered significant. All of the statistical analyses were performed using IBM SPSS for Windows (version 27.0; IBM Corporation).

### 2.5 Ethical considerations

The study was conducted in accordance with the relevant ethical standards^72^ and responsible research practice guidelines.^73^ Any necessary permits, registrations or licences for using the various instruments were obtained. The study was approved by the Institutional Review Board 16.9.2019 (OUKA/8626/07.01.04.02/2019). All eligible participants were given detailed written information about the study's purpose and objectives, as well as assurances regarding anonymity, confidentiality and the voluntary nature of participation. The researchers’ contact information was also provided so that prospective participants could ask additional questions if they wished. Completing and returning the anonymous questionnaire was considered to constitute informed consent for participation in the study. The data were collected, processed and stored without any identifying information. Thus, further ethical approval was not required.

## 3 RESULTS

### 3.1 Sample characteristics

The study sample consisted of 125 patients. The mean age of the participants was 68.5 years (SD = 10.7), with a range of 38 to 93 years. Well over half (59%) of the respondents were women, while 41% were men. Half (51%) had tertiary education, 20% had secondary education, and 29% had only completed basic education. All of the sample characteristics are shown in Table 1.

**Table 1:** Sample characteristics (mean, SD, range)

Characteristics (n = 125)	n (%)
Age Mean (SD). Median (range): 68.53 (10.720) 69 (38‐93)	
≤ 64 y	39 (31.2)
65‐74 y	49 (39.2)
≥75 y	35 (28)
Missing	2 (1.6)
Gender	
Female	74 (59.2)
Male	51 (40.8)
Education	
Primary education	36 (28.8)
Secondary education (high school/vocational education)	25 (20.0)
Tertiary education	64 (51.2)
Employment status	
Employed	11 (8.8)
Unemployed or long‐term sick leave	5 (4.0)
Retired (for various reasons)	109 (87.2)
Marital status	
Single	13 (10.4)
Married / In a registered partnership	79 (63.2)
Divorced	20 (16.0)
Widowed	13 (10.4)
Living situation	
Alone	36 (28.8)
With spouse/partner	63 (50.4)
With Spouse/partner and child/children	22 (17.6)
Something other	4 (3.2)

The participants had an average of four chronic conditions (range: 2‐13), with a wide variety of conditions and diseases being reported. The most common types of chronic physical conditions were hypertension (74% of the sample), diabetes (63%), coronary artery disease (27%), asthma (27%) and arrhythmia (24%). Depression was reported by 10% of the participants. In addition to the conditions mentioned above, 42% of the participants were obese.

### 3.2 The importance and realization of patient participation

The mean reported importance of PP was 4.32 (SD = 0.46; range: 3.15‐5.00), with 79% of the respondents assessing participation to be very important or extremely important (4 ≤ M ≤ 5). The subscale concerning importance of PP that received the highest rating was *Information and knowledge* (M = 4.58; SD = 0.41), while *Involvement of family* was assessed as least important (M = 3.77; SD = 1.34, Table 2).

**Table 2:** Summary of results of FI‐PPRQ scales for importance and realization (Mean, SD, range, Cronbach's alpha)

Dimension	Subscale (number of items)	N	Mean (SD)	Observed range mean^a^	Mean <3%	Mean ≥4%	Cronbach's Alpha
Importance	Information and knowledge (4)	124	4.58 (0.41)	3.25‐5.00	0	94.3	0.70
	Respect and integrity (4)	124	4.39 (0.57)	2.75‐5.00	0.8	83.1	0.82
	Motivation and encouragement (5)	124	4.33 (0.48)	3.00‐5.00	0	81.5	0.78
	Planning and decision making (4)	124	4.23 (0.61)	3.00‐5.00	0	68.5	0.84
	Involvement of family (2)	123	3.77 (1.03)	1.00‐5.00	12.2	54.5	0.85
Realization	Respect and integrity (4)	124	3.97 (0.80)	1.50‐5.00	9.7	63.7	0.90
	Information and knowledge (4)	124	3.92 (0.77)	1.50‐5.00	8.1	62.1	0.91
	Planning and decision making (4)	124	3.72 (0.86)	1.25‐5.00	12.9	41.9	0.92
	Motivation and encouragement (5)	124	3.62 (0.86)	1.20‐5.00	16.1	44.4	0.93
	Involvement of family (2)	122	2.53 (1.34)	1.00‐5.00	57.4	22.1	0.93

Note

Likert scale for importance: 1 = not at all important. 2 = slightly important. 3 = important. 4 = very important. 5 = extremely important.

Likert scale for realization: 1 = never. 2 = seldom. 3 = sometimes. 4 = often. 5 = always

SD = standard deviation.

^a^ Theoretical range mean 1‐5.

The mean reported realization of PP was 3,67 (SD = 0.73; range: 1.68‐5.00) on a scale in which a score of 3 indicated ‘sometimes’ and a score of 4 indicated ‘often’. About a third (32%) of respondents experienced that participation had been implemented somewhere between the ‘often’ and ‘always’ levels (4 ≤ M ≤ 5). The subscales concerning the realization of PP that received the highest ratings were *Respect and integrity* (M = 3.97; SD = 0.80) and *Information and knowledge* (M = 3.92; SD = 0.77), whereas *Involvement of family* received the lowest ratings (M = 2.53; SD = 1.34, Table 2).

### 3.3 Active participation and associated factors

Almost all of the respondents agreed that they had asked certain questions about their condition and care during health‐care encounters; more specifically, 45.6% of the respondents totally agreed, 51.8% agreed, while only a few (2.6%) disagreed with the statement. In terms of expressing views and opinions, 23.2% of the respondents totally agreed, 58.9% agreed, and 17.9% disagreed with the provided statement. However, both items have several missing cases, that is 8.8% and 10.4%, respectively ( 5). Higher patient activation and adherence were significantly positively associated with active participation during care encounters, that is patients’ manners regarding asking questions (*P* = .012 and *P* = .015, respectively) and expressing views/opinions (*P* = .030 and *P* = .040, respectively, Table 3).

**Table 3:** Patients’ active participation in health‐care encounters and associations with patient‐related factors (n, %, or mean, SD)

Factor	Asking questions Disagree‐agree‐totally agree	Expressing opinions / preferences Disagree‐agree‐totally agree
Total sample	n = 114 3 (2.6%)‐59 (51.8%)‐52 (45.6%)	n = 112 20 (17.9%)‐66 (58.9%)‐26 (23.2%)

Possible related categorical factors	n (%) *P* ^a^	n (%) *P* ^a^
Gender: female/ male	NS	NS
Education: primary/ secondary / tertiary	NS (*P* = .082 (FFH))	NS
Perceived health: poor/ moderate/ good	NS	NS
Perceived functional ability: poor/ moderate/ good	NS	NS

Possible related continuous factors	Mean (SD) *P* ^b^	Mean (SD) *P* ^b^
Age	NS	NS
Number of conditions	NS	NS
Patient activation	55.00 (18.1)‐52.18 (12.0)‐60.49 (13.7) .012 (ANOVA)	50.22 (10.8)‐55.62 (12.5)‐61.24 (14.9) .030 (ANOVA)
Adherence to care	3.86 (0.1)‐3.50 (0.4)‐3.70 (0.2) .015 (K‐W T)	3.46 (0.4)‐3.59 (0.3)‐3.71 (0.3) .040 (K‐W T)

^a^ Test: Fisher‐Freeman‐Halton exact test (FFH) or chi‐square test (χ^2^), as appropriate.

^b^ Kruskal‐Wallis test (K‐W T) or ANOVA, as appropriate.

### 3.4 Influences of patient‐related factors on subscales describing the importance of participation

As demonstrated in Table 4, certain patient characteristics (age, gender, perceived health, perceived functional ability, patient activation and active participation) significantly affected the scores of various subscales related to the importance of patient participation. Age was found to significantly influence *Respect and integrity* (*P* = .028), with older patients less likely to consider this aspect as highly important. Gender significantly influenced *Planning and decision making, Information and knowledge, Motivation and encouragement, and Involvement of family* (*P*‐values .002‐.020), as females evaluated the importance of each of these aspects higher than males. Perceived health and perceived functional ability were both connected with *Respect and integrity* (both *P* = .002) and *Motivation and encouragement* (*P* = .035 and *P* = .007, respectively); patients with good perceived health and good functional ability scored these aspects higher than their counterparts with lower perceived health and/or functional ability. Patient activation level affected each of the subscales, and patients with high activation considered each aspect more important to PP than those with low activation (*P* = .000‐.028). Also, patients’ active participation was significantly associated with almost all of the subscales; more specifically, the patients who totally agreed with items concerning asking questions and expressing views/opinions gave significantly higher scores to most of the aspects related to the importance of PP implemented by HCPs (*P*‐values between .009‐.039 and .000‐.010, respectively) than other patients.

**Table 4:** Connections of patient‐related factors with importance of patient participation (Mean, SD, *P*)

Factor	Patient participation (FI‐PPRQ)				
	Respect and integrity	Planning and decision making	Information and knowledge	Motivation and encouragement	Involvement of family
	Mean (SD)	Mean (SD)	Mean (SD)	Mean (SD)	Mean (SD)
	*P*‐value	*P*‐value	*P*‐value	*P*‐value	*P*‐value
Age					
≤64 y	4.58 (0.53)				
65‐74 y	4.33 (0.61)				
≥75 y	4.24 (0.53)				
	.028	NS	NS	NS	NS
Gender					
Female		4.34 (0.59)	4.68 (0.38)	4.44 (0.46)	3.97 (1.02)
Male		4.07 (0.61)	4.46 (0.42)	4.18 (0.48)	3.45 (. 96)
	NS	.02	.002	.003	.005
Educational level					
Primary					
Secondary					
Tertiary					
	NS	NS	NS	NS	NS
Number of conditions					
2‐3					
4‐5					
6 or more					
	NS	NS	NS	NS	NS
Perceived health					
Poor	4.40 (0.59)			4.32 (0.45)	
Moderate	4.16 (0.62)			4.20 (0.48)	
Good	4.56 (0.47)			4.45 (0.46)	
	.002	NS	NS	.035	NS
Perceived functional ability					
Poor	4.37 (0.58)			4.25 (0.46)	
Moderate	4.09 (0.66)			4.15 (0.47)	
Good	4.54 (0.48)			4.46 (0.46)	
	.002	NS	NS	.007	NS
Patient activation level					
Low	4.24 (0.67)	4.03 (0.60)	4.47 (0.49)	4.15 (0.45)	3.55 (0.96)
High	4.53 (0.44)	4.40 (0.59)	4.67 (0.35)	4.51 (0.41)	4.00 (0.1.03)
	.013	.003	.023	<.001	.028
Adherence to chronic care					
Poor					
Adequate					
Good					
	NS	NS	NS (.076)	NS (0.89)	NS
Active participation: asking questions in HC					
Disagree		3.83 (0.63)	4.58 (0.38)	4.20 (0.20)	3.67 (0.29)
Agree		4.09 (0.64)	4.47 (0.47)	4.21 (0.49)	3.50 (0.97)
Totally agree		4.42 (0.62)	4.71 (0.34)	4.49 (0.45)	4.00 (1.08)
	NS	.01	.012	.009	.039
Active participation: expressing views/opinions in HC					
Disagree	4.21 (0.64)	3.83 (0.64)		4.13 (0.50)	3.30 (0.92)
Agree	4.36 (0.59)	4.22 (0.59)		4.30 (0.48)	3.64 (1.02)
Totally agree	4.69 (0.39)	4.60 (0.46)		4.61 (0.38)	4.27 (0.98)
	.01	<.001	NS (.069)	.002	.003

Likert scale: 1 = not at all important. 2 = slightly important. 3 = important. 4 = very important. 5 = extremely important.

The one‐sample t test for pairwise comparisons. One‐way ANOVA for three groups

SD = standard deviation.

Significant at *P* < .05.

NS = non‐significant (*P* > .05).

### 3.5 Influences of patient‐related factors on subscales describing the realization of participation

As with the importance of patient participation, Table 5 shows that numerous patient characteristics (number of conditions, perceived health, perceived functional ability, patient activation, adherence and active participation) influence the perceptions of realization of PP in a statistically significant manner. The number of chronic conditions was significantly associated with the score for *Planning and decision making* (*P* = .006), as patients with 4‐5 conditions felt that PP was implemented more often than others. Perceived health and perceived functional ability both significantly influenced the *Respect and integrity* (*P* = .025 and *P* = .015, respectively), *Information and knowledge* (*P* = .026 and *P* = .006, respectively) and *Motivation and encouragement* (*P* = .010 and *P* = .004, respectively) subscales; patients with good perceived health reported better experiences of the implementation of PP than patients with low perceived health and/or functional ability. Patients with high activation levels perceived that PP—across all subscales—was more often realized than did patients with low activation (*P* = .000‐.008). Adherence to care was significantly positively associated with the reported degree to which PP was implemented related to Information and knowledge (*P* = .033) and Motivation and encouragement (*P* = .030). Finally, patients who totally agreed to the active participation question related to asking questions rated the realization of *Planning and decision making* (*P* = .042) higher than other patients, whereas patients who totally agreed that they expressed opinions during care encounters rated the realization of *Involvement of family* (*P* = .034) higher than other patients.

**Table 5:** Connections of patient‐related factors with realization of patient participation (Mean, SD, *P*)

Factor	Patient participation (FI‐PPRQ)				
	Respect and integrity	Planning and decision making	Information and knowledge	Motivation and encouragement	Involvement of family
	Mean (SD)	Mean (SD)	Mean (SD)	Mean (SD)	Mean (SD)
	*P*‐value	*P*‐value	*P*‐value	*P*‐value	*P*‐value
Age					
≤64 y					
65‐74 y					
≥75 y					
	NS	NS	NS	NS	NS
Gender					
Female					
Male					
	NS	NS	NS	NS	NS
Educational level					
Primary					
Secondary					
Tertiary					
	NS	NS	NS	NS	NS
Number of conditions					
2‐3		3.60 (1.00)			
4‐5		4.08 (0.64)			
6 or more		3.47 (0.60)			
	NS	.006	NS (.081)	NS	NS
Perceived health					
Poor	3.75 (0.61)		3.69 (0.70)	3.17 (0.76)	
Moderate	3.80 (0.90)		3.77 (0.80)	3.58 (0.81)	
Good	4.17 (0.74)		4.11.73)	3.82 (0.88)	
	.025	NS	.026	.01	NS
Perceived functional ability					
Poor	3.76 (0.71)		3.73 (0.68)	3.25 (0.69)	
Moderate	3.72 (0.88)		3.64 (0.86)	3.44 (0.89)	
Good	4.15 (0.75)		4.12 (0.71)	3.85 (0.85)	
	.015	NS (.057)	.006	.004	NS
Patient activation level					
Low	3.76 (0.76)	3.45 (0.85)	3.70 (0.76)	3.35 (0.79)	2.19 (1.14)
High	4.18 (0.76)	3.97 (0.78)	4.18 (0.65)	3.94 (0.78)	2.96 (1.46)
	.008	.002	.001	<.001	.005
Adherence to chronic care					
Poor			3.48 (1.07)	3.14 (0.84)	
Adequate			3.72 (0.88)	3.37 (0.86)	
Good			4.02 (0.68)	3.74 (0.84)	
	NS (.066)	NS	.033	.03	NS
Active participation: asking questions in HC					
Disagree		3.75 (0.66)			
Agree		3.53 (0.90)			
Totally agree		3.94 (0.80)			
	NS	.042	NS	NS	NS
Active participation: expressing views/opinions in HC					
Disagree					2.10 (1.15)
Agree					2.36 (1.24)
Totally agree					3.06 (1.66)
	NS	NS	NS	NS	.034

Likert scale: 1 = never. 2 = seldom. 3 = sometimes. 4 = often. 5 = always.

The one‐sample *t* test for pairwise comparisons. One‐way ANOVA for three groups

SD = standard deviation.

Significant at *P* < .05.

NS = non‐significant (*P* > .05).

### 3.6 Factors explaining patient's perceptions of patient participation

#### 3.6.1 Importance of total patient participation

The general linear model revealed that gender (*P* = .002), perceived health (*P* = .048), perceived functional ability (*P* = .052), patient activation (*P* = .000), adherence (*P* = .032) and active participation: both asking questions (*P* = .005) and expressing views/opinions (*P* = .000) were significant explanatory factors for the importance of PP in univariate analyses. Moreover, female patients considered participation more important than male patients. Patients with good perceived health and functional ability provided the highest ratings for the importance of patient participation, followed by patients with poor perceived health and functional ability, while patients with moderate perceived health and functional ability rated the importance of PP the lowest. Patient activation and adherence were both positively associated with perceptions of the importance of participation. Also, patients who totally agreed with the provided active participation statements about asking questions and expressing opinions considered participation implemented by HCPs to be more important than other patients (Table 6).

**Table 6:** General linear model for importance of patient participation (β, 95% CI)

Explanatory factor	Univariable model		Multivariable model 1		Multivariable model 2	
	β (95% CI)	*P* ^b^	Adjusted β (95% CI)^a^	*P* ^b^	Adjusted β (95% CI)^a^	*P* ^b^
Age (continuous factor)	−0.003 (−0.011‐0.005)	.457	−0.002 (−0.009‐0.006)	.66	0.000 (−0.008‐0.009)	.906
Gender						
Female	0.262 (0.102‐0.421)	.002	0.296 (0.117‐0.476)	.002	0.326 (0.142‐0.510)	.001
Male	ref.		ref.		ref.	
Education		.503		.132		.166
Primary	−0.045 (−0.236‐0.146)	.644	0.154 (−055‐0.362)	.146	0.163(−0.48‐0.375)	.127
Secondary	−0.127 (−0.341‐0.087)	.244	−0.101 (−0.333‐0.131)	.388	−0.075 (−0.296‐0.146)	.501
Tertiary	ref.		ref.		ref.	
Number of conditions (continuous factor)	0.010 (−0.031‐0.052)	.62	−0.025 (−0.070‐0.020)	.277	‐0.023 (−0.070‐0.024)	.34
Perceived health		.048		.006		
Poor	−0.036 (−0.261‐0.189)	.754	0.080 (−0.187‐0.346)	.552		
Moderate	‐0.219 (−0.396‐−0.041)	.016	−0.274 (−0.474‐−0.073)	.008		
Good	ref.		ref.			
Perceived functional ability		.052				.113
Poor	−0.088 (−0.296‐0.119)	.4			−0.125 (−0.351‐0.101)	.275
Moderate	−0.241 (−0.435‐−0.047)	.015			−0.270 (−0.479‐−0.062)	.012
Good	ref.				​ ref.	
Patient activation (PAM) (continuous factor)	0.008 (0.001‐0.016)	<.001	0.009 (0.002‐0.015)	.034	0.007 (0.000‐0.014)	.08
Adherence (ACDI) (continuous factor)	0.251 (0.022‐0.480)	.032	0.084 (−191‐0.360)	.544	0.048 (−0.221‐0.381)	.722
Asking questions		.005		.019		
Disagree	−0.345 (−0.870‐0.179)	.195	−0.404 (−1.003‐0.194)	.182		
Agree	−0.274 (−0.442‐−0.106)	.002	−0.261 (−0.460‐−0.063)	.011		
Totally agree	ref.		ref.			
Expressing views/ opinions		<.001				.002
Disagree	−0.552 (−0.808 ‐−0.297)	<.001			−0.386 (−0.680‐−0.092)	.011
Agree	−0.319 (−0.518 ‐−0.121)	.002			−0.373(−0.585‐−0.162)	.001
Totally agree	ref.				ref.	
			R‐squared = 0.368	<.001	R‐squared = 0.368	<.001

Β = regression coefficient for one‐unit increase in continuous factors and mean difference for categorical factors; CI = confidence interval.

^a^ Adjusted for other variables included in the model.

^b^ The Bonferroni correction was used in post hoc comparisons.

In the case of *multivariate analysis*, because perceived health and perceived functional ability, as well as asking questions and expressing views/opinions, were strongly correlated with each other, these variables were included in different multivariate models. The multivariate models, which were adjusted for all other variables included in the model, showed that in Model 1 (*P* = .000), including eight factors, the factors that significantly influenced the perceived importance of PP were gender (*P* = .002), perceived health (*P* = .006), patient activation (*P* = .034) and asking questions (*P* = .019), and the model explained 36.,8% of the variance for importance of patient participation. In Model 2 (*P* = .000), including eight factors, gender (*P* = .001) and expressing views/opinions (*P* = .002) remained significant and explained 36.8% of the variance for importance of patient participation (Table 6).

### 3.7 Realization of total patient participation

The general linear model revealed that perceived health (*P* = .032), perceived functional ability (*P* = .007), patient activation (*P* = .000) and adherence (*P* = .048) were significantly associated with the realization of PP in *univariate analyses*. Patients with good perceived health and functional ability reported the highest extent of the realization of participation, while patients with poor perceived health had the worst experiences of patient participation. Patients who adhered to their care felt that PP was better implemented than patients who did not completely adhere to their care. Patient activation was positively associated with patients’ experiences of the realization of participation. Two multivariate models, which both included eight variables and were adjusted for other variables in the model, revealed that patient activation (*P* = .000) significantly influences the realization of patient participation. Model 1 (*P* = .008) explained 27.6%, and Model 2 (*P* = .004) explained 29.6% of the variation in the realization of PP (Table 7).

**Table 7:** General linear model for realization of patient participation (β, 95% CI)

	Univariable model		Multivariable model 1		Multivariable model 2	
	β (95% CI)	*P* ^b^	Adjusted β (95% CI)^a^	*P* ^b^	Adjusted β (95% CI)^a^	*P* ^b^
Age (continuous factor)	‐0.003 (−0.015‐0.009)	.606	0.006 (−0.007‐0.018)	.378	0.009 (−0.004‐0.023)	.152
Gender						
Female	‐0.029 (−0.294‐0.236)	.830	0.068 (−0.228‐0.365)	.647	0.056 (−0.237‐0.349)	.702
Male	ref.		ref.		ref.	
Education		.953		.908		.788
Primary	0.005 (−0.300‐0.311)	.311	0.065 (−0.279‐0.409)	.709	0.095 (−0.242‐0.432)	.575
Secondary	0.062 (−0.281‐0.404)	.404	0.065 (−0.317‐0.448)	.734	0.96 (‐0.257‐0.448)	.591
Tertiary	ref.		ref.		ref.	
Number of conditions (continuous factor)	−0.013 (−0.080‐0.053)	.693	−0.056 (−0.131‐0.019)	.143	−0.056 (−0.131‐0.019)	.143
Perceived health		.032		.553		
Poor	−0.439 (−0.799‐−0.078)	.017	−0.024 (−0.416‐0.464)	.915		
Moderate	−0.270 (−0.554‐0.015)	.063	−0.158 (−0.490‐0.173)	.344		
Good	ref		ref.			
Perceived functional ability		.007				.251
Poor	−0.430 (−0.757‐−0.102)	.011			0.027 (−0.379‐0.433)	.895
Moderate	−0.395 (−0.702‐−0.088)	.012			−0.284 (−0.659‐0.091)	.135
Good	ref.	.			ref.	
Patient activation (PAM) (continuous factor)	0.024 (0.014‐0.034)	<.001	0.028 (0.016‐0.040)	<.001	0.028 (0.016‐0.041)	<.001
Adherence (ACDI) (continuous factor)	0.497 (0.131‐0.863)	.008	−0.149 (−0.603‐0.306)	.517	−0.164 (−0.594‐0.266)	.449
Asking questions		.157		.898		
Disagree	−0.167 (−1.028‐0.694)	.702	−0.169 (−1.159‐0.819)	.743		
Agree	−0.270 (−0.546‐0.006)	.055	−0.055 (−0.382‐0.273)	.74		
Totally agree	ref.		ref.			
Expressing views/ opinions		.405				.628
Disagree	−0.278 (−0.710‐0.155)	.206			0.222 (−0.248‐0.691)	.35
Agree	−0.185 (−0.522‐0.152)	.28			0.045 (−0.292‐0.382)	.791
Totally agree	ref.				ref.	
			R‐squared = 0.276	.008	R‐squared = 0.296	.004

β = regression coefficient for one‐unit increase in continuous factors and mean difference for categorical factors; CI = confidence interval.

^a^ Adjusted for other variables included in the model.

^b^ The Bonferroni correction was used in post hoc comparisons.

## 4 DISCUSSION

This study has provided new knowledge about multimorbid patients’ perceptions of the importance of PP and the degree to which it is implemented by HCPs, as well as to what extent the patients themselves actively participate in health‐care encounters in Finnish primary health‐care settings. In addition, the presented research provided insight into which specific factors influence patients’ perceptions of patient participation.

The study revealed that respondents consider PP to be an important aspect of their care, which is an important finding and also consistent with previous findings in chronic primary health‐care patients.^20^, ^59^, ^74^ However, ratings regarding the importance of participation demonstrated some variability, and some subscales were considered to be more important than others. For example, patients rated *Information and knowledge* as the most important aspect of participation, with the item ‘*The patient should receive information provided by professionals in a way she/ he can understand’* receiving the highest score. This is understandable given that multimorbid patients have a great need for information and that information can be confusing, and even contradictory, when several HCPs are involved.^27^, ^28^, ^32^ The *involvement of the family* subscale received the lowest scores and also showed the greatest variation. This may be explained by the fact that not all patients have, or want to involve, family members. However, family‐centred care has been described as a central component of PP^7^, ^61^ and patient‐centred care of multimorbid patients^29^; thus, relatives should always be able to get involved if the patient so wishes.

Perceptions of the extent to which PP was realized showed substantial variation, with responses ranging from always to seldom, if ever. This variability was also noticeable across all of the subscales, which suggests that perceptions of the degree to which participation is implemented are highly patient‐specific. This means that adequate PP may be challenging to achieve with multimorbid patients. This may be because PP is known to be a challenge to achieve in general,^3^, ^19^ and complex multimorbidity care is likely to make it more complicated, but even more imperative. In this study, the *Respect and integrity* and *Information and knowledge* subscales received the highest ratings. This is important, as respect has been reported to be essential for patient participation,^3^, ^74^ as well as a prerequisite for adequate information exchange. As a such, HCPs can influence PP and empowerment through their supporting actions.^3^ This is consistent with the findings of a focus group study across eight European countries that concentrated on the perspectives of multimorbid patients; that is, being approached and supported holistically by HCPs is vital to a good care process.^36^

According to the results, differences in perceptions of participation were associated with varied patient‐related factors. Gender was found to affect patients’ perceptions regarding the importance of participation but did not affect the perceptions regarding the extent to which PP was realized. Female patients gave significantly higher scores to almost all subscales for importance of PP than male patients, and further, female gender was also a significant exploratory factor for the total importance of participation in multivariate analyses. These results agree with previous findings that women are more likely to feel that PP is important,^59^, ^61^ have a stronger preference for involvement in medical decisions,^75^, ^76^ be more interested in health‐related information^77^ and declare a more active attitude towards treatment than men.^78^

Perceived health and functional ability were found to significantly impact patients’ perception of both the importance and realization of participation, but did not exert a considerable effect on active participation. Patients with both good and poor perceived health and functional ability found *Respect and integrity*, along with *Motivation and encouragement*, more important than patients with moderate perceived health and functional ability. Patients with good perceived health and functional ability, on the other hand, felt that these two subscales, along with *Information and knowledge*, to be best implemented by professionals. This may suggest that multimorbid patients with severe problems in health and/or functional ability feel that their need for respect, access to information and encouragement is not adequately addressed. A previous study reported somewhat similar results; that is, respondents with poor perceived health gave worse reviews of HCP communication than other patients.^79^ Poor perceived health has also been found to predict lower overall satisfaction with health care.^80^

The number of conditions a patient was afflicted by significantly influenced *Planning and decision making*, as patients with 4‐5 diseases perceived that PP was realized more often than patients suffering from fewer or more conditions. An explanation may be that they have had enough recurring health‐care visits to experience realization of PP, but not yet too many confusing diseases, as it is known that planning and decision making tend to become more complex as number of diseases related to multimorbidity increases.^24^

Patient activation, that is patient's knowledge, skills and confidence, as well as the belief in the importance of their own role in managing their own health, had a predictably strong effect on participation. Patients with high activation provided positive ratings on all of the subscales related to the importance and realization of participation and were those who showed more active participation, that is asked questions and expressed their opinion more often than patients with lower activation. It was also revealed to be an explanatory factor for patients’ perceptions of PP. These findings are supported by reports that patient activation affects experiences of health services. In chronic patients, patient activation was found to be negatively associated with the reporting of care coordination problems ^33^ and perceived barriers during medical consultation,^81^ and positively associated with the perceived quality of interpersonal exchanges with physicians, fairness in the treatment process,^82^ persistence in asking questions when the patient did not understand something,^83^ taking an active role in medical decisions^84^ and perceived care experience.^85^

Adherence was found to be associated with several subscales regarding the importance and realization of patient participation. Adherence was also positively related to active participation, consistent with previous findings in primary care suggesting that a patient's active participation is associated with treatment adherence.^86^ These findings may suggest that patients who demonstrate high activation, adherence and/or appreciation for the realization of PP may have more confidence and urgency to have HCPs respond to their needs. Previous research has shown that patients who actively communicate with HCPs will receive care that is more patient‐centred and informative^87^, ^88^; again, physician's communication style and degree of patient‐centredness were identified to be strong predictors of active participation.^15^ In this study, almost all of the respondents reported asking certain questions, but approximately one‐sixth disagreed that they expressed their views and opinions during health‐care encounters. Both of these questions included numerous missing answers, which was not observed for other questions; hence, the respondents may have found it difficult to assess their own behaviour.

This study has some strengths and limitations. The questionnaires used in this study relied on self‐reporting, the questionnaires were distributed by HCPs during appointments with patients in many units, and it is not known how many forms were distributed. These characteristics introduce some risk of bias. However, the research—which focused on patients’ perceptions—applied validated instruments to collect data. The way forms were distributed afforded respondents the opportunity to evaluate recent encounters with the experience still clearly in mind. Furthermore, the questionnaire allowed them to assess the care at a time that was most convenient to them. To ensure honesty, the questionnaires were returned anonymously to the researcher. However, the chosen method of data collection proved to be challenging, as the COVID‐19 epidemic emerged shortly after the start of data collection and sharply reduced the number of non‐urgent primary care appointments, including those for the chronically ill patient population examined here. This may partly explain why the sample size remained quite small. Nevertheless, the sample includes patients with a wide variety of diseases and conditions and is representative of numerous age and other sociodemographic groups. This study was conducted in Finland, and as such, the findings may not be generalizable to other populations elsewhere. The employed cross‐sectional design was used to ascertain associations between the studied factors, but does not enable any analyses of causality.

## 5 CONCLUSIONS

Multimorbid patients generally found PP to be important, but reported widely varying degrees to which PP had been implemented by HCPs. Several patient‐related factors were found to affect patients’ perceptions of PP, as well as to explain patients’ perceptions of PP implemented by HCPs in both dimensions (importance and realization). Most respondents actively participated during health‐care encounters, which was positively associated with patient activation and adherence to care, as well as perceived importance of PP. Patient activation seemed to be strongly intertwined with active participation and perceptions of how supportive HCPs were of PP. Poor perceived health and functional ability seemed to predispose patients to worse perceptions towards PP, a finding that should be investigated in more detail.

The results suggest that PP should be individualized. Care for multimorbid patients should promote PP and active communication in health‐care encounters, which might also have the potential to improve patient activation and adherence to care. Moreover, high‐quality care is the result of both a patient's own actions and effective collaboration with professionals.

## CONFLICTING INTERESTS

The authors declared no potential conflicts of interest with respect to the research, authorship and/or publication of this article.

## Data Availability

Data are available on request from the authors.
